# Considerations on the Haigis formula: Are better outcomes possible with tuning?

**DOI:** 10.1111/aos.17491

**Published:** 2025-03-29

**Authors:** Achim Langenbucher, Nóra Szentmáry, Jascha Wendelstein, Alan Cayless, Benj Fassbind, Peter Hoffmann

**Affiliations:** ^1^ Department of Experimental Ophthalmology Saarland University Homburg, Saar Germany; ^2^ Dr. Rolf M. Schwiete Center for Limbal Stem Cell and Aniridia Research Saarland University Homburg, Saar Germany; ^3^ Department of Ophthalmology Semmelweis University Budapest Budapest Hungary; ^4^ Department of Ophthalmology Ludwig‐Maximilians‐University Munich Germany; ^5^ School of Physical Sciences The Open University Milton Keynes UK; ^6^ Augen‐ und Laserklinik Castrop‐Rauxel Castrop‐Rauxel Germany

**Keywords:** formula constant optimization, formula constants, formula upgrade, Haigis formula, IOL power formula, vergence calculation

## Abstract

**Purpose:**

To design a vergence‐based lens power formula based on the classical Haigis formula for better outcomes while retaining the original formula architecture.

**Methods:**

Four new formula variants (A–D) incorporating a sum of segments correction for axial length, harmonic mean of corneal radii instead of arithmetic mean (all variants), and differing combinations of lower keratometer index (C, D) and an additional term (a3) representing the lens thickness in the effective lens position (B, D) were assessed in an analysis based on four datasets of IOLMaster 700 biometric data for eyes treated with the Hoya Vivinex lens (dataset 1), Alcon SA60AT lens (2), Johnson & Johnson ZCB00 lens (3), and the Bausch & Lomb MX60 lens (4). All parameters (formula constants and keratometer index) were calculated by nonlinear iterative optimisation techniques for minimising the root mean squared prediction error (RMSPE). Performance was assessed in terms of the final RMSPE.

**Results:**

All four variants showed reductions in RMSPE ranging from 2.8% to 12.6% over the original Haigis formula. For each of the four datasets, variants B and D (with the additional a3 constant) performed better in this respect than variants A and C. In all four cases, variants C and D (with the adjusted keratometer index) performed slightly better than A and B, respectively.

**Conclusion:**

Although not amenable to statistical analysis, the % improvements in RMSPE would appear to be clinically relevant. However, the benefit has to be proven in a prospective multicentric study with a large sample size.

## 1 BACKGROUND

Building on the classical vergence formulae for intraocular lens power calculations, the third‐generation formulae SRK/T (Retzlaff et al., 1990; Sanders et al., 1990), Hoffer Q (Hoffer, 1981, 1993; Hoffer & Savini 2020) and Holladay 1 (Holladay et al., 1988) were further refined with the introduction of the Haigis formula (Haigis et al., 2000). Developed in the 1990s and published in 2000, the fourth‐generation Haigis formula uses 3 ‘a’ constants and typically shows a good performance over the entire range of biometric values.

All four formulae are based on a pseudophakic model eye containing three refractive surfaces: a thin lens spectacle correction, the cornea, and the intraocular lens (IOL) implant itself (Langenbucher et al., 2021; Langenbucher, Hoffmann, et al., 2023). Compared with the other formulae, the Haigis formula uses a lower keratometer index of nK = 1.3315 (Holladay et al., 1988) for the conversion of corneal radius given in mm to corneal power (for comparison: SRK/T: nK = 1.333 (Retzlaff et al., 1990; Sanders et al., 1990), Hoffer Q: nK = 1.3375 (Hoffer, 1981, 1993; Hoffer & Savini 2020), Holladay: nK = 4/3 (Holladay et al., 1988)). Haigis uses the arithmetic mean of the corneal radii in the flat meridian (R1) and in the steep meridian (R2) (Holladay et al., 1988), whereas the other formulae use the arithmetic mean of the keratometric powers (K) in the flat (K1) and the steep meridians (K2) (Hoffer, 1981, 1993; Hoffer & Savini 2020; Holladay et al., 1988; Retzlaff et al., 1990; Sanders et al., 1990).

The effective lens position (ELP), which refers to a ‘fictitious’ axial position of the thin lens implant with respect to the corneal front surface plane, is derived from a multiple linear regression with an intercept parameter (a0) and two further terms representing the phakic anterior chamber depth (ACD) (with a weight a1), and the axial length (AL) (with a weight a2). In the simplified version of the Haigis formula, preset values of a1 = 0.4 and a2 = 0.1 are used (Aristodemou et al., 2011; Behndig et al., 2014; El‐Khayat & Tesha, 2021; Galvis et al., 2013), and the intercept parameter a0 is tuned to consider the characteristics of the IOL. This simplified version is mostly used where there are not enough reliable clinical data for optimisation of all three formula constants. However, it is well known that the preset a1 and a2 values are not optimal for modern lens models, and that optimising all three formula constants a0, a1 and a2 improves the performance of the Haigis formula significantly, especially at the tails of the biometric parameter distributions (e.g. for very long or short eyes) (Langenbucher et al., 2022; Langenbucher et al., 2023b).

The development of the Haigis formula was to a certain extent linked to the development of optical biometry with the IOLMaster (Carl‐Zeiss‐Meditec, Jena, Germany), which was first launched in 1999. This first generation of the IOLMaster measured keratometry at 6 points located on a circle of diameter ~ 3.2 mm (R1, R2 and the orientation of the flat corneal meridian) together with the axial length (AL) and the anterior chamber depth (ACD) measured from the corneal apex to the lens front apex. All of these parameters are used in the Haigis formula. Newer optical biometers derive additional biometric data, such as the central thickness of the lens (LT), the central corneal thickness, the horizontal corneal diameter, and optionally the curvature of the corneal back surface (Savini et al., 2020), raising the interesting possibility of enhancing or extending the Haigis formula with the inclusion of one or more of these newly available measures (LT in this study).

The study had two principal aims: first, to develop a vergence‐based lens power formula based on the classical Haigis formula with enhancements in terms of calculating the mean corneal radius, considering a sum of segments concept for calibration of axial length measures, including the lens thickness measure, and a variation of the keratometric index. Second, to evaluate the performance of the upgraded lens power formula compared with the performance of the classical Haigis formula based on four clinical datasets containing biometric data, the power of the implanted lens and the postoperative subjective refraction.

## 2 METHODS

### 2.1 Datasets for our study

Four datasets were analysed in this retrospective study. The first dataset contains measurements from 888 eyes (489 right and 397 left eyes) treated with the 1‐piece hydrophobic aspherical (aberration correcting) monofocal intraocular Vivinex lens (Hoya Surgical, Singapore). The second dataset contains measurements from 821 eyes (415 right and 406 left eyes) treated with the 1‐piece hydrophobic spherical monofocal intraocular SA60AT lens (Alcon, Fort Worth, USA). The third dataset contains measurements from 613 eyes (314 right and 299 left eyes) treated with the 1‐piece hydrophobic aspherical (aberration correcting) monofocal intraocular ZCB00 lens (Johnson & Johnson Vision, Jacksonville, USA). The fourth dataset contains measurements from 467 eyes (278 right and 189 left eyes) treated with the 1‐piece hydrophobic aspherical (aberration free) monofocal intraocular MX60 lens (Bausch & Lomb, Rochester, USA). Each dataset contained only one eye per individual, randomly selected in cases where data of both eyes were available. Eyes with a history of ocular surgery and eyes with any pathology affecting the refractive outcome after cataract surgery (ectatic corneal diseases, zonular weakness, uncontrolled ocular hypertension or glaucoma, and retinal pathologies) were discarded from the datasets at the clinical centre prior to transfer. All eyes in the datasets showed a visual acuity of at least 0.2 logMAR at the postoperative follow‐up examination to ensure a reliable postoperative refraction.

All eyes underwent cataract surgery at the Augen‐ und Laserklinik Castrop‐Rauxel, Castrop‐Rauxel, Germany. The local Institutional Review Board (Ärztekammer des Saarlandes, registration number 157/21) provided a waiver for this study, and patient informed consent was not required for this study. The data were transferred to us in an anonymised fashion, precluding back‐tracing of the patient.

The anonymised datasets contained preoperative biometric data from the IOLMaster 700 (Carl‐Zeiss‐Meditec, Jena, Germany), including axial length AL, anterior chamber depth ACD measured from the corneal epithelium to the anterior apex of the crystalline lens, the central thickness of the crystalline lens LT, the corneal front surface radius measured in the flat and steep meridians R1 and R2, the labelled refractive power of the intraocular lens IOLP and the spherical equivalent of manual subjective refraction as documented 5–12 weeks after cataract surgery by an experienced optometrist at a refraction lane distance of 6 m. To ensure the reliability of the postoperative refraction, the dataset included only data with a postoperative Snellen decimal visual acuity of 0.8 (20/25 Snellen lines) or higher.

### 2.2 Preprocessing of the data

The anonymised Excel data (.xlsx‐format) were imported into MATLAB (Matlab 2022b, MathWorks, Natick, USA) for further processing with a custom data processing code. Four variants of a modified Haigis formula were prepared for investigation. Two changes were included in all four variants: firstly, in contrast to the original Haigis formula in which R is derived from the arithmetic mean of R1 and R2, all four variants of our formula used the harmonic mean R = 2·R1·R2/(R1 + R2). This change was made because the harmonic mean refers to the spherical equivalent power of the spherocylindrical corneal surface. With corneal astigmatism, the arithmetic mean always overestimates R (or underestimates corneal power), and the difference between the harmonic and the arithmetic mean −(R1 − R2)^2^/(2·(R1 + R2)) increases with the difference between the steep and flat radii. Second, we implemented the sum of segments corrections for the AL measures as described by Cooke in 2019 (Cooke & Cooke, 2019a, 2019b) in all four variants of the modified formula.

Four variants (A–D) of the modified formula were assessed against the classical Haigis formula (Haigis et al., 2000) which was used as a reference:

Variant A modifies the classical Haigis formula with the addition of the two features previously described: the use of the harmonic mean for R1 and R2, and the sum of segments correction for the AL. This variant uses a keratometer index of nK = 1.3315 and a linear regression for the ELP prediction with (ELP = a0 + a1·ACD + a2·AL).

Variant B includes an additional linear term relating to the LT in the ELP prediction (ELP = a0 + a1·ACD + a2·AL + a3·LT).

Variant C uses the ELP calculation of variant A together with an optimised keratometer index.

Finally, Variant D includes the ELP prediction of variant B (with the additional term a3·LT) together with the optimised keratometer of variant C.

This means that for variant A we optimised three parameters (a0/a1/a2), for variants B and C, we optimised 4 parameters (a0/a1/a2/a3 and a0/a1/a2/nK respectively), and for variant D, we optimised 5 parameters (a0/a1/a2/a3/nK).

For the classical Haigis formula and all four variants of our formula, and for each of the four datasets, we first derived the optimised formula constants a0/a1/a2 (versions A and C) and a0/a1/a2/a3 (versions B and D) together with the optimised keratometer index nK (versions C and D) using the iterative nonlinear sequential quadratic programming algorithm (SQP) as described in previous papers and using the formula prediction error (PE, defined as the difference between the formula prediction and the achieved postoperative SEQ) as the target parameter. Constant optimisation was performed for root mean squared PE. A step size tolerance of 1e−10 and a function tolerance of 1e−12 were used as the stopping criteria for the algorithm (Langenbucher et al., 2021, 2022; Langenbucher, Hoffmann, et al., 2023; Langenbucher et al., 2023a, 2023b). The distributions of the predicted ELP and of the PE were calculated for all five formulae (original Haigis and all four variants A, B, C, and D) using each of the four datasets (1, 2, 3 and 4).

In the next step, to test the robustness of the formula constants (Langenbucher, Hoffmann, et al., 2023; Langenbucher et al., 2023a) and the keratometer index (Langenbucher et al., 2023b), we used a bootstrapping strategy where all parameters in the dataset (biometric data, IOLP and SEQ were sampled NB = 5000 times with replacement). For each bootstrap sample, we calculated the optimised formula constants (either a0/a1/a2 for formula variants A and C or a0/a1/a2/a3 for formula variants B and D) and nK (for variants C and D), and from all of these NB = 5000 bootstrapped formula constants and keratometer indices, we derived the standard deviation and the limits of the 95% confidence intervals.

In the last step, we used the combined dataset (combining all readings from datasets 1–4) to derive a common keratometer index nKC to be used in clinical practice. With this nKC, the formula constants a0a1/a2 or a0/a1/a2/a3 were again adjusted for dataset 1 to dataset 4 to be used together with nKC in our new vergence formulae, and the root mean squared prediction error was extracted.

### 2.3 Statistical analysis and data presentation

Data are listed descriptively in terms of the arithmetic mean, standard deviation (SD), median and the lower and upper boundaries of the 95% confidence interval (2.5% and 97.5% quantiles). The distributions of the predicted ELP and the prediction error PE for the original Haigis formula and for the four formula variants A, B, C and D for each of the four datasets are shown using cumulative distribution function plots (CDF).

## 3 RESULTS

Table 1 lists the descriptive data for the relevant preoperative biometric measures together with the labelled lens power and the spherical equivalent of postoperative refraction for the four clinical datasets considered in our analysis. Table 2 lists the optimised formula constants (a0/a1/a2 for the original Haigis formula and formula variants A and C and a0/a1/a2/a3 for formula variants B and D) together with the preset nK (original Haigis formula and formula variants A and B) and optimised nK (formula variants C and D) for datasets 1–4. The penultimate column of the table details the root mean squared PE, which has been used as an optimisation metric, and the last column lists the % improvement in RMSPE as compared with the original formula. The data imply that for datasets 1 and 3, the gain in performance is systematically larger when including the LT term in the ELP prediction (formula variants A vs. B and C vs. D) as compared with the gain in performance for datasets 2 and 4. The table also presents that the optimised nK value (formula versions C and D, range from 1.3254 to 1.3310) is always lower compared with the preset nK value (nK = 1.3315) used in the original Haigis formula and formula variants A and B.

**TABLE 1:** Descriptive statistics of the four datasets in terms of mean, standard deviation (SD), median, and the lower (quantile 2.5%) and upper (quantile 97.5%) boundaries of the 95% confidence interval.

Explorative description	AL in mm	ACD in mm	LT mm	R1 in mm	R2 in mm	R in mm	IOLP in dpt	SEQ in dpt
Dataset 1: Hoya Vivinex (*N* = 888)								
Mean	24.0922	3.1848	4.6215	7.8589	7.6731	7.7641	20.6377	−0.5624
SD	1.4034	0.4072	0.4499	0.2824	0.2748	0.2681	3.7215	0.9246
Median	23.9001	3.1847	4.5932	7.8467	7.6723	7.7626	21.0000	−0.2500
Quantile 2.5%	21.6749	2.3715	3.7563	7.3335	7.1328	7.2686	12.0000	−2.5000
Quantile 97.5%	27.3536	3.9439	5.5194	8.4291	8.2153	8.3013	27.5000	0.5000
Dataset 2: Alcon SA60AT lens (*N* = 821)								
Mean	23.1467	3.0434	4.6219	7.7807	7.6176	7.6977	22.7369	−0.4780
SD	1.5107	0.3986	0.4120	0.2721	0.2742	0.2656	4.5956	0.7152
Median	23.1800	3.0260	4.6100	7.8100	7.6400	7.7297	22.5000	−0.2500
Quantile 2.5%	20.4510	2.3060	3.8200	7.1903	7.0303	7.1052	13.5000	−2.6250
Quantile 97.5%	26.4297	3.8180	5.4200	8.2698	8.1000	8.1798	33.0000	0.5000
Dataset 3: J & J ZCB00 lens (*N* = 613)								
Mean	23.4558	3.1755	4.6388	7.7625	7.5870	7.6729	22.3418	−0.5137
SD	1.3958	0.4084	0.4235	0.2822	0.2694	0.2636	3.9449	0.7897
Median	23.3800	3.1900	4.6400	7.7500	7.5700	7.6699	22.0000	−0.2500
Quantile 2.5%	20.7713	2.3350	3.7100	7.2448	7.0535	7.1747	14.0000	−2.5000
Quantile 97.5%	26.9535	3.9817	5.4487	8.3600	8.1200	8.2209	31.0875	0.5000
Dataset 4: B&L MX60 lens (*N* = 467)								
Mean	24.4409	3.2443	4.6362	7.8301	7.6576	7.7424	198 340	−0.7586
SD	2.0697	0.3521	0.3895	0.2474	0.2534	0.2431	5.3005	0.8855
Median	24.0400	3.2600	4.6600	7.8000	7.6400	7.7290	21.0000	−0.5000
Quantile 2.5%	21.4387	2.5859	3.8817	7.4300	7.2317	7.3435	4.0000	−2.7500
Quantile 97.5%	31.3795	3.9441	5.4195	8.3900	8.1065	8.2075	29.0000	0.2500

*Note*: Parameters listed are: Axial length (AL), external phakic anterior chamber depth measured from the corneal front apex to the front apex of the crystalline lens (ACD), central lens thickness (LT), corneal radii in the flat (R1) and steep (R2) meridians, the harmonic mean of R1 and R2 (R), refractive power of the intraocular lens implant (IOLP), and the spherical equivalent power achieved 4–12 weeks after cataract surgery (SEQ).

**TABLE 2:** Formula constants a0/a1/a2/a3 and keratometer index nK for the original Haigis formula (Haigis) and the four variants of the modified formula.

Dataset	Formula	a0	a1	a2	a3	nK	RMS PE in dpt	Performance gain in %
Dataset 1: Hoya Vivinex (*N* = 888)	Haigis	−0.6853	0.3417	0.2029		1.3315	0.3714	–
	Variant A	1.0608	0.3849	0.1237		1.3315	0.3540	4.69
	Variant B	0.0510	0.5808	0.1027	0.1928	1.3315	0.3414	8.08
	Variant C	1.7776	0.3620	0.0827		1.3280	0.3486	6.14
	Variant D	0.8468	0.5622	0.0597	0.1847	1.3281	0.3363	9.45
Dataset 2: Alcon SA60AT lens (*N* = 821)	Haigis	−0.7607	0.2925	0.2016		1.3315	0.4157	–
	Variant A	0.7769	0.3700	0.1269		1.3315	0.4040	2.81
	Variant B	−0.0106	0.4903	0.1147	0.1521	1.3315	0.3965	4.62
	Variant C	1.7336	0.3525	0.0706		1.3270	0.3974	4.40
	Variant D	0.9594	0.4744	0.0572	0.1529	1.3269	0.3896	6.28
Dataset 3: J & J ZCB00 lens (*N* = 613)	Haigis	−1.0702	0.2962	0.2312		1.3315	0.4105	–
	Variant A	0.5903	0.3732	0.1509		1.3315	0.3890	5.24
	Variant B	−0.6674	0.5705	0.1272	0.2567	1.3315	0.3666	10.69
	Variant C	1.8329	0.3403	0.0785		1.3254	0.3779	7.94
	Variant D	0.4684	0.5259	0.0696	0.2340	1.3265	0.3588	12.59
Dataset 4: B&L MX60 lens (*N* = 467)	Haigis	−0.3897	0.3224	0.1928		1.3315	0.3871	–
	Variant A	1.6137	0.3740	0.1014		1.3315	0.3656	5.55
	Variant B	1.1730	0.4771	0.0860	0.1026	1.3315	0.3623	6.41
	Variant C	1.7591	0.3695	0.0938		1.3310	0.3653	5.63
	Variant D	1.3542	0.4768	0.0762	0.1019	1.3309	0.3620	6.48

*Note*: All four variants A–D implemented the corneal radius as the harmonic mean of the radii in the flat and steep meridians and used the Cooke sum of segments correction for axial length. Variant A uses constants a0/a1/a2 with preset value nK = 1.3315, variant B uses constants a0/a1/a2/a3 with preset value nK = 1.3315, variant C uses constants a0/a1/a2 and optimised nK, and variant D uses constants a0/a1/a2/a3 and optimised nK. RMS PE refers to the root mean squared formula prediction error. The last column refers to the relative lowering of the RMS PE of formula variants A/B/C/D with respect to the original Haigis formula.

**FIGURE 1:**
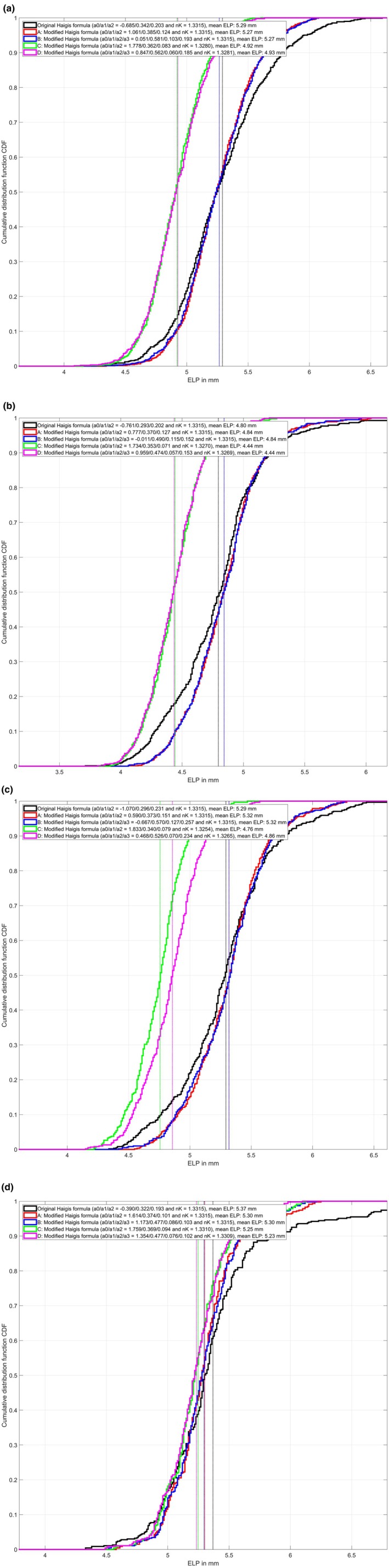
Cumulative distribution function (CDF) for the predicted effective lens position ELP for the original Haigis formula and formula variants A, B, C, and D. The ELP is derived from a linear superposition: ELP = a0 + a1·ACD + a2·AL (original Haigis formula and formula variants A and C) or ELP = a0 + a1·ACD + a2·AL + a3·LT (formula variants B and D). (a) Refers to dataset 1, (b) to dataset 2, (c) to dataset 3, and (d) to dataset 4. In each case, the formula constants and keratometer indices (nK) used in the formula versions are shown in the corresponding figure legends. The flatter CDF curve for the original Haigis formula indicates a larger variation in the predicted ELP as compared with formula variants A–D. The mean ELP is indicated with dashed vertical lines in the graph and noted in the figure legends.

Figure 1 shows the cumulative distribution function CDF for the predicted ELP from either the linear superposition a0 + a1·ACD + a2·AL (variants A and C) or a0 + a1·ACD + a2·AL + a3·LT (variants B and D) for dataset 1 (Figure 1a), dataset 2 (Figure 1b), dataset 3 (Figure 1c) and dataset 4 (Figure 1d). For all four datasets, there is no systematic difference in the ELP distribution when comparing formula variants A and B or C and D, but in both of the variants with optimised keratometer index nK (C and D) the ELP gives systematically lower values compared with the ELP in the variants with preset nK (A and B). However, the CDF curve for the original Haigis formula is flatter for all four datasets, indicating a larger variation in the ELP prediction compared with formula variants A–D. The corresponding mean values for the ELP are indicated with vertical dashed lines and listed in the figure legends.

**FIGURE 2:**
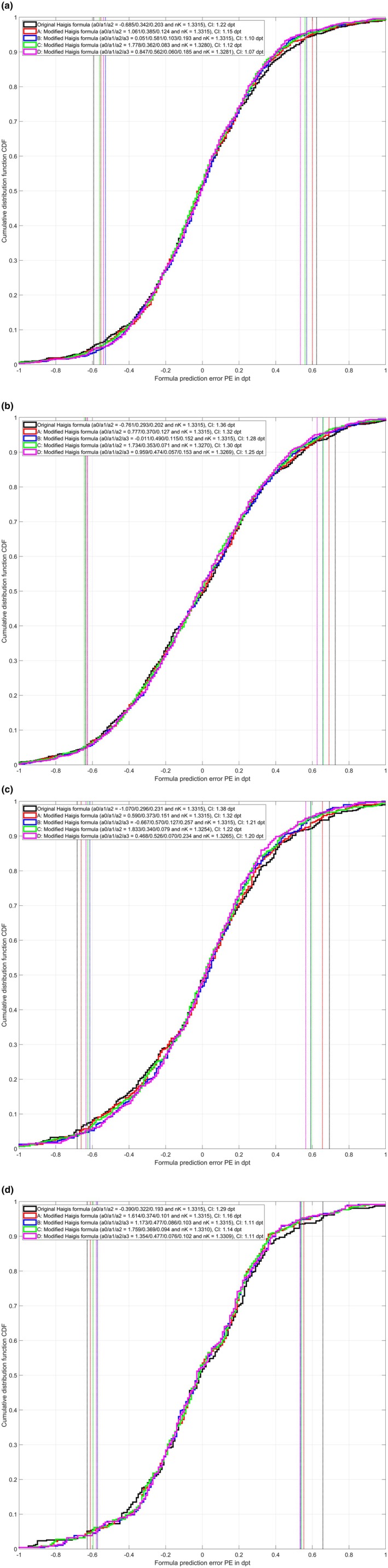
Cumulative distribution function (CDF) for the formula prediction error PE for the original Haigis formula and formula variants A (formula constants a0/a1/a2 and preset nK = 1.3315), B (formula constants a0/a1/a2/a3 and preset nK = 1.3315), C (formula constants a0/a1/a2 and adjusted nK) and D (formula constants a0/a1/a2/a3 and adjusted nK). (a) Refers to dataset 1, (b) to dataset 2, (c) to dataset 3, and (d) to dataset 4. The lower and upper limits of the 95% confidence interval are shown with vertical dashed lines, and the width of the 90% confidence interval is noted in the figure legends (CI).

Figure 2 shows the cumulative distribution function CDF for the formula prediction error PE for the original Haigis formula and the formula variants A, B, C and D for dataset 1 (Figure 2a), dataset 2 (Figure 2b), dataset 3 (Figure 2c) and dataset 4 (Figure 2d). For the PE derived from the optimised formula constants (either a0/a1/a2 for the original Haigis formula and formula variants A and C or a0/a1/a2/a3 for formula variants B and D) and preset nK (original Haigis formula and formula variants A and B) or optimised nK (formula variants C and D) the lower and the upper limits of the 90% confidence intervals are shown in the graph (vertical dashed lines) and the widths of the 90% confidence intervals are listed in the legends.

The results for the formula constant and keratometer index uncertainties in terms of lower and upper limits of the 95% confidence intervals and the standard deviations derived from bootstrapping resampling are listed in Table 3 for all four datasets and for formula variants A, B, C and D plus the original Haigis formula. The results show that in general the uncertainty in a0 is systematically larger compared with the uncertainty in a1, which is again larger compared with the uncertainty in a2. In the formulae with four formula constants (variants B and D) the uncertainty in a3 is quite similar to the uncertainty in a1. It is important to mention that the standard deviation for nK (variants C and D) ranging from 0.0005 to 0.0007 is much lower compared with the corresponding standard deviation in any of the formula constants.

**TABLE 3:** Formula constant and keratometer index uncertainties in terms of lower and upper boundaries of the 95% confidence interval (2.5% and 97.5% quantiles) and standard deviation (SD) derived from NB = 5000 bootstrap samples with replacement.

Formula variant	Dataset 1: Hoya Vivinex (*N* = 888)	Dataset 2: Alcon SA60AT lens (*N* = 821)	Dataset 3: J &J ZCB00 lens (*N* = 613)	Dataset 4: B&L MX60 lens (*N* = 467)
	2.5%/97.5% quantile/SD	2.5%/97.5% quantile/SD	2.5%/97.5% quantile/SD	2.5%/97.5% quantile/SD
Haigis formula				
a0	−0.9329/−0.4395/0.1268	−1.0094/−0.5077/0.1282	−1.4052/−0.7293/0.1726	−0.8357/0.0012/0.2133
a1	0.3046/0.3801/0.0188	0.2498/0.3341/0.0216	0.2511/0.3422/0.0236	0.2575/0.3760/0.0307
a2	0.1915/0.2145/0.0060	0.1888/0.2144/0.0066	0.2159/0.2476/0.2308	0.1744/0.2141/0.01021
Variant A				
a0	0.0827/1.2948/0.1175	0.5347/1.0111/0.1250	0.2599/0.8908/0.1609	1.1837/1.9877/0.2072
a1	0.3784/0.3881/0.0029	0.3262/0.3814/0.0160	0.3253/0.3875/0.0180	0.3373/0.3802/0.0103
a2	0.1146/0.1330/0.0047	0.1170/0.1397/0.0059	0.1380/0.1675/0.0075	0.0872/0.1196/0.0083
Variant B				
a0	−0.2642/0.3485/0.1573	−0.3088/0.3123/0.1605	−1.0104/−0.3059/0.1811	0.6960/1.5873/0.2284
a1	0.5369/0.6250/0.0222	0.4368/0.5404/0.0264	0.5132/0.6273/0.0288	0.4088/0.5471/0.0358
a2	0.0914/0.1147/0.0059	0.1021/0.1279/0.0066	0.1127/0.1424/0.0076	0.0654/0.1079/0.0109
a3	0.1586/0.2294/0.0059	0.1099/0.1930/0.0211	0.2126/0.2997/0.0220	0.0466/0.1587/0.0286
Variant C				
a0	1.5018/2.0609/0.1422	1.3582/2.0419/0.1741	1.3904/2.2148/0.2078	1.2801/2.1975/0.2336
a1	0.3510/0.3700/0.0049	0.3076/0.3603/0.0134	0.2929/0.3588/0.0174	0.3354/0.3789/0.0106
a2	0.0698/0.0956/0.0066	0.0563/0.0915/0.0089	0.0603/0.1028/0.0108	0.0750/0.1162/0.0104
nK	1.3272/1.3289/0.0005	1.3257/1.3283/0.0007	1.3241/1.3268/0.0007	1.3299/1.3322/0.0006
Variant D				
a0	0.4680/1.2122/0.1869	0.5755/1.3663/0.2033	0.0054/0.9335/0.2378	0.8138/1.8458/0.2625
a1	0.5182/0.6064/0.0223	0.4220/0.5260/0.0262	0.4717/0.5828/0.0288	0.4043/0.5470/0.0363
a2	0.0430/0.0754/0.0083	0.0369/0.0768/0.0102	0.0482/0.0913/0.0108	0.0537/0.1020/0.0122
a3	0.1501/0.2196/0.0174	0.1124/0.1939/0.0209	0.1918/0.2749/0.0213	0.0443/0.1567/0.0284
nK	1.3272/1.3290/0.0005	1.3256/1.3282/0.0007	1.3251/1.3278/0.0007	1.3290/1.3321/0.0006

*Note*: All four variants A–D implemented the corneal radius as the harmonic mean of the radii in the flat and steep corneal meridians and used the Cooke sum of segments correction for axial length measures. Variant A uses constants a0/a1/a2 with preset value nK = 1.3315, variant B uses constants a0/a1/a2/a3 with preset value nK = 1.3315, variant C uses constants a0/a1/a2 and optimised nK, and variant D uses constants a0/a1/a2/a3 and optimised nK. The confidence intervals and standard deviations for the formula constants and the keratometer index are listed for all 4 datasets.

For clinical use of the new vergence formula with a common keratometer index value, optimising nK for the entire dataset (combination of datasets 1–4) yields nKC = 1.3294. With this keratometer index nK, the new formula reads

$$\text{predicted IOLP}=\frac{1.336}{\mathrm{AL}-\mathrm{ELP}}-\frac{1}{\frac{1}{\frac{\mathrm{TR}}{1-0.012∙\mathrm{TR}}+\frac{329.4}{R}}-\frac{\mathrm{ELP}}{1336}}$$

for the predicted refractive power of the IOL with target refraction at the spectacle plane (TR) or

$$\text{predicted}\;\mathrm{SEQ}=\frac{1}{\frac{1}{\frac{1}{\frac{1}{\frac{1}{\frac{1336}{\mathrm{AL}-\mathrm{ELP}}}-\text{PIOL}}+\frac{\mathrm{AL}-\mathrm{ELP}}{1336}}-\frac{329.4}{R}}+0.012}$$

for the predicted refraction at the spectacle plane. The ELP could be calculated from a linear superposition with either three formula constants (ELP = a0 + a1·ACD + a2·AL) or with four formula constants including an LT term (ELP = a0 + a1·ACD + a2·AL + a3·LT). With the common nK = 1.3294, the adjusted formula constants for the four IOL types under test read:

Hoya Vivinex lens: a0/a1/a2 = 1.5021/0.3723/0.0984 or a0/a1/a2/a3 = 0.05444/0.5692/0.0761/0.1877; root mean squared PE: 0.3495 or 0.3371 dpt.

Alcon SA60AT lens: a0/a1/a2 = 1.2257/0.3623/0.1005 or a0/a1/a2/a3 = 0.4363/0.4829/0.0883/0.1524; root mean squared PE: 0.3993 or 0.3916 dpt.

Johnson & Johnson ZCB00 lens: a0/a1/a2 = 1.0215/0.3618/0.1259 or a0/a1/a2/a3 = −0.1894/0.5516/0.1030/0.2471; root mean squared PE: 0.3827 or 0.3614 dpt.

Bausch & Lomb MX60 lens: a0/a1/a2 = 2.2024/0.3528/0.0709 or a0/a1/a2/a3 = 1.8295/0.4760/0.0505/0.1003; root mean squared PE: 0.3681 or 0.3645 dpt.

## 4 DISCUSSION

Over the last 25 years, the Haigis formula as a classical 4th generation vergence formula has proven to be one of the best lens power formulae for the entire range of biometric parameters (Chang et al., 2023; Darcy et al., 2020; Voytsekhivskyy et al., 2021, 2023). Especially with a serious optimisation of the constant triplet a0/a1/a2 trend errors, for example, over the axial length (Langenbucher et al., 2022; Langenbucher et al., 2023a, 2023b; Shrivastava et al., 2021; Zhang et al., 2019) are quite low compared with other formulae of the 80s and 90s. However, there are some elements in the formula which could easily be upgraded to enhance the performance of the formula. First, the corneal radius defined in the formula definition as the arithmetic mean of the radii in the flat and steep meridian should be replaced by the harmonic mean to be in line with the equivalent power of the cornea and consistent with all other formulae. Secondly, a sum of segments correction for the axial length measure could be included to avoid overestimation of the AL for long eyes (and underestimation for short eyes). Third, even though the keratometer index used in the Haigis formula is lower than that in all of the other formulae of this age, it is derived from the Gullstrand model eye and refers to the equivalent power derived from the Gullstrand formula. However, it is now known that the corneal front to back surface radius ratio defined in the Gullstrand model eye might overestimate the normal situation, and according to most of the modern schematic model eyes the back surface should be somewhat steeper than the 6.8 mm defined in the Gullstrand model (Jin et al., 2024; Langenbucher et al., 2021, 2022; Langenbucher, Hoffmann, et al., 2023; Langenbucher et al., 2023b). For this reason, we decided to include the keratometer index in our multiple parameter optimisation (formula variants C and D), resulting in a slightly lower keratometer index which is around 1.3294 for the entire dataset. The constant triplet used in the Haigis formula refers to the ELP prediction, and a0 is used as intercept and a1 and a2 as scaling for the ACD and AL in a linear regression setup. As we did not intend to change the principal architecture of the Haigis formula, we decided to keep the linear regression strategy for the ELP prediction, but to add one biometric parameter which we feel is of great relevance for the ELP prediction. We therefore added a term a3·LT to the ELP prediction with the intention of reducing the dependency of the classical Haigis formula on the stage of the lens opacification (Yesilkaya & Garip, 2023) or the thickness of the crystalline lens (Sella et al., 2024). As we know, the crystalline lens grows over time (Achiron et al., 2024) and especially with opacification the anterior pole moves forward while the posterior pole moves backward (Yesilkaya & Garip 2023). Therefore, our idea was to counterbalance the effect of decreasing ACD with the increase in LT.

The most relevant outcome from our study was that for all four datasets derived with four modern lenses on the market from different manufacturers, the purely data‐driven optimisation of the keratometer index yielded a value that is quite similar to that obtained from modern schematic model eyes (e.g. Liou & Brennan or Kooijman) when calculating corneal power with respect to the front apex plane. To keep the modifications of the Haigis formula simple and intuitive, we decided to use a common keratometer index (nK = 1.3294) derived from the entire dataset (including *N* = 2790 eyes). From Figure 1 we learn that reducing the value of nK systematically reduces the ELP, mostly in a range of around 0.35 to 0.50 mm. However, we also noticed that decreasing the nK does not simply shift the CDF curves to the left. This means that the distribution of the ELP is also changed. We feel that these lower values for the ELP are much closer to the anatomical IOL position, which can be directly measured with modern biometers or anterior segment tomographers (Sardari et al., 2023). The flatter CDF curves for the original Haigis formula (shown as black lines) indicate that the variation in predicted ELP is wider compared with formula variants A–D.

With the inclusion of the additional term for the ELP prediction (a3·LT term in formula variants B and D) we noticed that the formula performance is improved systematically for all four datasets. We observed the largest benefit in datasets 3 and 1, where aspherical lenses with a high and medium correction of spherical aberration were implanted, and much less benefit in datasets 2 and 4 where a spherical or aberration neutral lens was used. Overall, as with classical keratometer indices for conversion of corneal front surface radius to corneal power, the refractive power of the cornea is mostly overestimated and the ‘fictitious parameter’ ELP has to be increased to compensate (Aristodemou et al., 2011; Behndig et al., 2014; Langenbucher et al., 2021; Langenbucher et al., 2023b; Sardari et al., 2023). However, as this shift in ELP has a much larger effect on the formula predicted refraction in short eyes, which require high powered IOLs, we feel that a down‐adjustment of nK to a more realistic value is justified, even if we require new formula constants throughout. However, using the 3 or 4 constant version of the new Haigis formula 2.0 (variant C or D) is mostly a matter of taste: we feel that by including a term for LT (which could not be measured with optical biometry when the first IOLMaster has been launched) we could somehow reduce the effect of age (Achiron et al., 2024; Sella et al., 2024) or cataract stage dependency (Yesilkaya & Garip, 2023), but we have to deal with two different sets of formula constants: a constant triplet and a formula quadruplet.

From the bootstrapping results shown in Table 3 we learn that the intercept parameter a0 shows the largest variation (broadest 95% confidence interval and largest standard deviation), followed by a1 (and a3 for formula variants B and D) and a2. This is not surprising, as if we consider that a1 is scaled by the ACD (with a mean value of around 3.2 mm) and a2 is scaled by AL (with a mean value of around 23.7 mm) (and a3 is scaled by LT with a mean value of around 4.6 mm). This means that for example a small variation in a1, a3, and especially in a2 requires a large variation in a0 to compensate and produce the same ELP value.

For comparison of the classical Haigis formula and the variants A/B/C/D with a modern high‐performance formula, we optimised the A constant for the Cooke K6 formula on our four datasets using the identical optimisation strategy (minimisation of the root‐mean‐squared PE with nonlinear iterative SQP algorithm). For dataset 1/dataset 2/dataset 3/dataset 4, we read out an optimised A constant of 119.2260/118.7821/119.3801/119.1924 and a root‐mean‐squared PE of 0.3381/0.3933/0.3521/0.3556 dpt. Compared with the respective values shown in Table 2 especially formula variants B and D yield very similar performance values.

However, our study has some limitations: First, we wanted to keep the basic architecture of the Haigis formula unchanged. Therefore, the study was restricted to a linear superposition of terms for the ELP prediction, even though we are aware that nonlinear functions might show a better performance. Second, for the same reason, we restricted the analysis to a pseudophakic model eye with three refractive surfaces, even though we know that a thick lens model for the cornea might be more appropriate, especially with uncommon front‐toto‐back surface radii ratios (Jin et al., 2024) of the cornea, for example, after corneal refractive surgery (Langenbucher, Hoffmann, et al., 2023). And third, we used monocentric data with four lens models to derive the new vergence formula based on the classical Haigis formula. For generalisation, multicentric data with much larger sample sizes could help to validate the (purely data‐driven) keratometer index and the formula constant triplets or quadruplets.

In conclusion, this article shows a straightforward strategy to upgrade a classical vergence formula for calculation of intraocular lenses while keeping the internal structure of the formula unchanged. Adjusting the keratometer index and adding a term to the ELP to take into account the thickness of the crystalline lens improved the results in all four datasets. However, the gain in performance was not the same in all datasets, and the Hoya Vivinex and the Johnson & Johnson ZCB00 profited much more than the Alcon SA60AT and the Bausch & Lomb MX60 from the formula upgrade. The benefits of our upgraded Haigis 2.0 formula should be evaluated independently with a multicentric prospective study and a larger sample size.

## References

[aos17491-bib-0001] Achiron, A. , Yahalomi, T. , Biran, A. , Levinger, E. , Cohen, E. , Elbaz, U. et al. (2024) A comprehensive evaluation of 16 old and new intraocular lens power calculation formulas in pediatric eyes. Clinical Ophthalmology, 18, 2225–2238. Available from: 10.2147/OPTH.S470425 39135944 PMC11318601

[aos17491-bib-0002] Aristodemou, P. , Knox Cartwright, N.E. , Sparrow, J.M. & Johnston, R.L. (2011) Intraocular lens formula constant optimization and partial coherence interferometry biometry: refractive outcomes in 8108 eyes after cataract surgery. Journal of Cataract and Refractive Surgery, 37(1), 50–62. Available from: 10.1016/j.jcrs.2010.07.037 21183099

[aos17491-bib-0003] Behndig, A. , Montan, P. , Lundström, M. , Zetterström, C. & Kugelberg, M. (2014) Gender differences in biometry prediction error and intra‐ocular lens power calculation formula. Acta Ophthalmologica, 92(8), 759–763. Available from: 10.1111/aos.12475 24930806

[aos17491-bib-0004] Chang, P. , Qian, S. , Wang, Y. , Li, S. , Yang, F. , Hu, Y. et al. (2023) Accuracy of new‐generation intraocular lens calculation formulas in eyes with variations in predicted refraction. Graefe's Archive for Clinical and Experimental Ophthalmology, 261(1), 127–135. Available from: 10.1007/s00417-022-05748-w 35802204

[aos17491-bib-0005] Cooke, D.L. & Cooke, T.L. (2019a) Approximating sum‐of‐segments axial length from a traditional optical low‐coherence reflectometry measurement. Journal of Cataract and Refractive Surgery, 45(3), 351–354. Available from: 10.1016/j.jcrs.2018.12.026 30851808

[aos17491-bib-0006] Cooke, D.L. & Cooke, T.L. (2019b) A comparison of two methods to calculate axial length. Journal of Cataract and Refractive Surgery, 45(3), 284–292. Available from: 10.1016/j.jcrs.2018.10.039 30851805

[aos17491-bib-0007] Darcy, K. , Gunn, D. , Tavassoli, S. , Sparrow, J. & Kane, J.X. (2020) Assessment of the accuracy of new and updated intraocular lens power calculation formulas in 10 930 eyes from the UK National Health Service. Journal of Cataract and Refractive Surgery, 46(1), 2–7. Available from: 10.1016/j.jcrs.2019.08.014 32050225

[aos17491-bib-0008] El‐Khayat, A.R. & Tesha, P. (2021) Optimizing the intraocular lens formula constant according to intraocular lens diameter. International Journal of Ophthalmology, 14(5), 700–703. Available from: 10.18240/ijo.2021.05.09 34012884 PMC8077020

[aos17491-bib-0009] Galvis, V. , Tello, A. & Portorreal, J. (2013) Impact of constant optimization of formulae. Graefe's Archive for Clinical and Experimental Ophthalmology, 251(10), 2477–2478. Available from: 10.1007/s00417-013-2381-9 23695658

[aos17491-bib-0010] Haigis, W. , Lege, B. , Miller, N. & Schneider, B. (2000) Comparison of immersion ultrasound biometry and partial coherence interferometry for intraocular lens calculation according to Haigis. Graefe's Archive for Clinical and Experimental Ophthalmology, 238(9), 765–773. Available from: 10.1007/s004170000188 11045345

[aos17491-bib-0011] Hoffer, K.J. (1981) Intraocular lens calculation: the problem of the short eye. Ophthalmic Surgery, 12(4), 269–272. 7254770

[aos17491-bib-0012] Hoffer, K.J. (1993) The Hoffer Q formula: a comparison of theoretic and regression formulas. Journal of Cataract and Refractive Surgery, 19(6), 700–712. Available from: 10.1016/s0886-3350(13)80338-0 8271165

[aos17491-bib-0013] Hoffer, K.J. & Savini, G. (2020) Update on intraocular lens power calculation study protocols: the better way to design and report clinical trials. Ophthalmology, 128(11), e115–e120. Available from: 10.1016/j.ophtha.2020.07.005 32653457

[aos17491-bib-0014] Holladay, J.T. , Prager, T.C. , Chandler, T.Y. , Musgrove, K.H. , Lewis, J.W. & Ruiz, R.S. (1988) A three‐part system for refining intraocular lens power calculations. Journal of Cataract and Refractive Surgery, 14(1), 17–24. Available from: 10.1016/s0886-3350(88)80059-2 3339543

[aos17491-bib-0015] Jin, A. , Zhang, J. , Tan, X. , Jin, K. , Zhang, Y. , Han, X. et al. (2024) Effect of posterior keratometry on the accuracy of 10 intraocular lens calculation formulas: standard keratometry versus total keratometry. Graefe's Archive for Clinical and Experimental Ophthalmology, 262(6), 1829–1838. Available from: 10.1007/s00417-023-06367-9 38197993

[aos17491-bib-0016] Langenbucher, A. , Hoffmann, P. , Cayless, A. , Wendelstein, J. & Szentmáry, N. (2023) Limitations of constant optimization with disclosed intraocular lens power formulae. Journal of Cataract and Refractive Surgery, 50(3), 201–208. Available from: 10.1097/j.jcrs.0000000000001337 PMC10878441 37847110

[aos17491-bib-0017] Langenbucher, A. , Szentmáry, N. , Cayless, A. , Müller, M. , Eppig, T. , Schröder, S. et al. (2021) IOL formula constants: strategies for optimization and defining standards for presenting data. Ophthalmic Research, 64(6), 1055–1067. Available from: 10.1159/000514916 33530082 PMC8743903

[aos17491-bib-0018] Langenbucher, A. , Szentmáry, N. , Cayless, A. , Wendelstein, J. & Hoffmann, P. (2022) Strategies for formula constant optimisation for intraocular lens power calculation. PLoS One, 17(5), e0267352. Available from: 10.1371/journal.pone.0267352 35511906 PMC9071153

[aos17491-bib-0019] Langenbucher, A. , Szentmáry, N. , Cayless, A. , Wendelstein, J. & Hoffmann, P. (2023a) Evaluating intraocular lens power formula constant robustness using bootstrap algorithms. Acta Ophthalmologica, 101(3), e264–e274. Available from: 10.1111/aos.15277 36286335

[aos17491-bib-0020] Langenbucher, A. , Szentmáry, N. , Cayless, A. , Wendelstein, J. & Hoffmann, P. (2023b) Formula constant optimisation techniques including variation of keratometer or corneal refractive index and consideration for classical and modern IOL formulae. PLoS One, 18(2), e0282213. Available from: 10.1371/journal.pone.0282213 36827418 PMC9956664

[aos17491-bib-0021] Retzlaff, J.A. , Sanders, D.R. & Kraff, M.C. (1990) Development of the SRK/T intraocular lens implant power calculation formula. Journal of Cataract and Refractive Surgery, 16(3), 333–340. Available from: 10.1016/s0886-3350(13)80705-5 2355321

[aos17491-bib-0022] Sanders, D.R. , Retzlaff, J.A. , Kraff, M.C. , Gimbel, H.V. & Raanan, M.G. (1990) Comparison of the SRK/T formula and other theoretical and regression formulas. Journal of Cataract and Refractive Surgery, 16(3), 341–346. Available from: 10.1016/s0886-3350(13)80706-7 2355322

[aos17491-bib-0023] Sardari, S. , Khabazkhoob, M. , Jafarzadehpur, E. & Fotouhi, A. (2023) Comparison of intraocular lens power calculation between standard partial coherence interferometry‐based and Scheimpflug‐based biometers: the importance of lens constant optimization. Journal of Current Ophthalmology, 35(1), 42–49. Available from: 10.4103/joco.joco_32_23 37680291 PMC10481983

[aos17491-bib-0024] Savini, G. , Taroni, L. & Hoffer, K.J. (2020) Recent developments in intraocular lens power calculation methods‐update 2020. Annals of Translational Medicine, 8(22), 1553. Available from: 10.21037/atm-20-2290 33313298 PMC7729321

[aos17491-bib-0025] Sella, R. , Reitblat, O. , Durnford, K.M. , Pettey, J.H. , Olson, R.J. , Hahn, T.E. et al. (2024) The effect of patient age on some new and older IOL power calculation formulas. Acta Ophthalmologica, 102(5), e696–e704. Available from: 10.1111/aos.16621 38155407

[aos17491-bib-0026] Shrivastava, A.K. , Nayak, S. , Mahobia, A. , Anto, M. , Kacher, R. & Kumar, A. (2021) Optimizing lens constants specifically for short eyes: is it essential? Indian Journal of Ophthalmology, 69(9), 2293–2297. Available from: 10.4103/ijo.IJO_63_21 34427203 PMC8544056

[aos17491-bib-0027] Voytsekhivskyy, O.V. , Hoffer, K.J. , Savini, G. , Tutchenko, L.P. & Hipólito‐Fernandes, D. (2021) Clinical accuracy of 18 IOL power formulas in 241 short eyes. Current Eye Research, 46(12), 1832–1843. Available from: 10.1080/02713683.2021.1933056 34013799

[aos17491-bib-0028] Voytsekhivskyy, O.V. , Hoffer, K.J. , Tutchenko, L. , Cooke, D.L. & Savini, G. (2023) Accuracy of 24 IOL power calculation methods. Journal of Refractive Surgery, 39(4), 249–256. Available from: 10.3928/1081597X-20230131-01 37040214

[aos17491-bib-0029] Yesilkaya, E.C. & Garip, R. (2023) Accuracy of different lens power calculation formulas in patients with mature cataracts. Cureus, 15(10), e47053. Available from: 10.7759/cureus.47053 38021815 PMC10644268

[aos17491-bib-0030] Zhang, J.Q. , Zou, X.Y. , Zheng, D.Y. , Chen, W.R. , Sun, A. & Luo, L.X. (2019) Effect of lens constants optimization on the accuracy of intraocular lens power calculation formulas for highly myopic eyes. International Journal of Ophthalmology, 12(6), 943–948. Available from: 10.18240/ijo.2019.06.10 31236350 PMC6580219

